# *UEG* Journal and Impact on Clinical Practice

**DOI:** 10.1002/ueg2.12722

**Published:** 2024-11-30

**Authors:** Joost P. H. Drenth

**Affiliations:** ^1^ Amsterdam University Medical Center Amsterdam the Netherlands

This month, I will leave the *UEG* Journal after serving for 6 years as its Editor‐in‐Chief of the *UEG* Journal. Being Editor was a job that brought me intense joy. I thoroughly enjoyed the daily grind of reading papers and making decisions [1]. I was very fortunate to have a fantastic team of Associate Editors, quick, sharp, and critical and I indebted to them for their efforts to bring *UEG* Journal to greater heights. What sets *UEG* Journal really apart, is the cadre of trainee editors that we have within our fold since the beginning of my tenure [2]. The presence of these young professionals has changed the Journal on many levels [3]. Trainee editors are intimately involved in the editorial process cycle as they, among others, judge manuscripts, perform search engine optimization, and create visual abstracts [4]. They have been pivotal to the success of our endeavour [5, 6, 7, 8, 9, 10].

The Journal is the flagship of *UEG*, and since 2021 Wiley is our publisher. Wiley offers more than 20 other Journals that share Gastroenterology & Hepatology as their main subject matter. Within 3 years we have firmly established ourselves among one of the leading journals within their portfolio. As of 2021, we switched from a subscription model to fully open access. This strategy comes with article publication charges that is carried by authors, the author's institution, or their research funder. We are aware of the difficulties that this may bring to authors, and Wiley partners with *UEG* to develop strategies to mitigate these costs for our authors. The upside is that critical research information from the *UEG* Journal becomes freely available to readers at no cost. Being hosted by a professional publishing house such as Wiley, allows further growth of the Journal in a rapidly changing publishing world.

Back to the content that we publish. During my tenure, I have witnessed a profound change in types of manuscripts. We have witnessed a move from retrospective single centre studies to prospective multicentre studies that possess more robust outcomes and have a better impact on clinical practice. Just to mention a few: the Italian PITER collaboration, Dutch Initiative on Crohn's and Colitis, the Hungarian Pancreatic Study Group, the Grupo de Estudos da Doença Inflamatória Intestinal, and the Dutch T1 CRC working group [11, 12, 13, 14, 15]. We continue to applaud these national collaborative initiatives and look forward to judge their future work.

This year marks the publication of the 3rd special issue [16, 17]. We started this path in 2022 with a complete issue devoted to inflammatory bowel diseases, followed by an issue on liver diseases and this issue revolves around pancreatic disorders. The idea is to bring readers with topical reviews by experts that summarize the state‐of‐art in the field. We are constantly evaluating our endeavours and we listen to you, the reader, with the aim to serve you best.

One important part of our offerings are guidelines with we offer in conjunction with *UEG* *'s* quality and care committee [18, 19]. They maintain a Standards & Guidelines Repository and coordinate initiatives to promote and disseminate appropriate standards of GI care. The *UEG* Journal regularly publish these important documents. Most guidelines are comprehensive, well researched and referenced documents, but the challenge is to find that important recommendation you need in a lengthy text. We are investigating how we can provide an attractive digestible format to you that helps you to have an impact in clinical practice.

From 2025, I will move on to different pastures but I will continue to serve *UEG* (albeit in a different capacity) as its vice‐president. I am indebted to my current team of Associate Editors, Alexander Meining, Christophe Moreno, Fernando Magro, Yasuka Maeda, Mao Ren, and Daniel Keszthelyi. They made work fun with their quick and witty responses and appreciation of each Editor's routines (I know who is up early). Editing is teamwork and I am indebted to all of them. A last word for Albrecht Neesse who will steer this ship in the coming years. We count ourselves lucky to have you aboard as the incoming Editor‐in‐Chief of the *UEG* Journal. As a smart thinker, you are going to make a difference for the Journal, enjoy the voyage just as I did, or better, more.

## Conflicts of Interest

The author declares no conflicts of interest.

## Data Availability

The data that support the findings of this study are available on request from the corresponding author. The data are not publicly available due to privacy or ethical restrictions.
